# Intestinal Phosphorus Absorption in Chronic Kidney Disease

**DOI:** 10.3390/nu10101364

**Published:** 2018-09-23

**Authors:** Elizabeth R. Stremke, Kathleen M. Hill Gallant

**Affiliations:** Department of Nutrition Science, Purdue University, West Lafayette, IN 47907, USA; hillgallant@purdue.edu

**Keywords:** phosphorus, phosphate, chronic kidney disease, CKD, absorption, dietary phosphorus

## Abstract

Chronic kidney disease (CKD) affects approximately 10% of adults worldwide. Dysregulation of phosphorus homeostasis which occurs in CKD leads to development of CKD-Mineral Bone Disorder (CKD-MBD) and contributes to increased morbidity and mortality in these patients. Phosphorus is regulated by multiple hormones (parathyroid hormone (PTH), 1,25-dihyxdroxyvitamin D (1,25D), and fibroblast growth factor 23 (FGF23)) and tissues (kidney, intestine, parathyroid glands, and bone) to maintain homeostasis. In health, the kidneys are the major site of regulation for phosphorus homeostasis. However, as kidney function declines, the ability of the kidneys to adequately excrete phosphorus is reduced. The hormonal changes that occur with CKD would suggest that the intestine should compensate for impaired renal phosphorus excretion by reducing fractional intestinal phosphorus absorption. However, limited studies in CKD animal models and patients with CKD suggest that there may be a break in this homeostatic response where the intestine fails to compensate. As many existing therapies for phosphate management in CKD are aimed at reducing absolute intestinal phosphorus absorption, better understanding of the factors that influence fractional and absolute absorption, the mechanism by which intestinal phosphate absorption occurs, and how CKD modifies these is a much-needed area of study.

## 1. Introduction: The Importance of Managing Phosphorus Homeostasis in CKD

### 1.1. CKD is a Major Health Problem that Disrupts Phosphorus Metabolism

Chronic kidney disease (CKD) affects approximately 26 million American adults in the United States and approximately 1 in 3 people are at risk [1]. CKD is a disease of progressive decline in renal function that includes disruption of normal phosphorus homeostasis as the kidney loses its ability to regulate phosphorus by urinary excretion. Notably, abnormalities in phosphorus homeostasis have been shown to occur early in CKD progression due to disease-mediated alterations in the hormonal regulators of phosphorus metabolism, well before clinical hyperphosphatemia is observed [2]. This is a central component of CKD-mineral bone disorder (CKD-MBD), which is a condition characterized by (1) abnormalities in laboratory values related to calcium and phosphorus metabolism, (2) increased vascular calcifications, and (3) bone disease, all of which interact and contribute to increased risk for cardiovascular events, bone fragility fractures, and death [3]. In fact, the leading cause of death in patients with CKD is cardiovascular disease, not kidney failure [4]. Higher serum phosphorus itself has been associated with disease progression in CKD patients [5]. Because disturbed phosphorus metabolism is considered an instigating factor in the development and progression of CKD-MBD, current therapies for CKD-MBD include controlling phosphorus metabolism. Much of this is aimed at limiting absolute intestinal phosphorus absorption since the failing kidney is not a suitable point of intervention. 

### 1.2. Reducing Dietary Phosphorus Intake or Absorption is a Strategy to Combat the Effects of CKD on Phosphorus Homeostasis

Limiting dietary phosphorus intake and intestinal absorption is a central strategy to reduce the phosphorus burden on the kidney with the goal to prevent disease progression as well as mitigate the increased risk of CKD-MBD and associated morbidity and mortality [6,7]. The National Kidney Foundation Kidney Disease Outcomes Qualities Initiative’s (KDOQI) clinical guidelines for CKD state that dietary phosphorus should be restricted to 800 to 1000 mg/day for CKD patients based on stage of disease and presence of elevated serum phosphate or parathyroid hormone (PTH) [1]. While this intake level still exceeds the 700 mg/day recommended daily allowance for most adults [8], it is still restrictive when compared to the average American phosphorus intake of ~1500 mg/day [9,10]. However, because phosphorus-restricted diets are difficult to consistently maintain and because intestinal phosphorus fractional absorption is relatively efficient (~60–70% is absorbed from a mixed diet) [11,12], phosphorus binder medications are frequently prescribed to be taken with meals to decrease bioaccessibility of phosphorus and, as a result, decrease the absolute intestinal phosphorus absorption in CKD patients [3,13]. Another emerging approach is to pharmacologically target the fractional absorptive capacity of the intestine, by inhibiting phosphate transporters or by changing the intestinal epithelial tight junctions to be less permeable to phosphate ions [14]. However, important knowledge gaps exist related to mechanisms of intestinal phosphorus absorption and how various factors influence it, particularly in the context of CKD. This suggests that treatment approaches have yet to be optimized. Thus, a recent report from a National Kidney Foundation symposium identified intestinal phosphorus absorption as a key research area [15] needed to improve treatment of CKD-MBD. This review will provide an overview of phosphorus absorption, its role in phosphorus homeostasis, and what is currently known regarding phosphorus absorption in the context of CKD. 

## 2. Overview of the Physiologic Mechanisms Controlling Phosphorus Homeostasis

**Figure 1:**
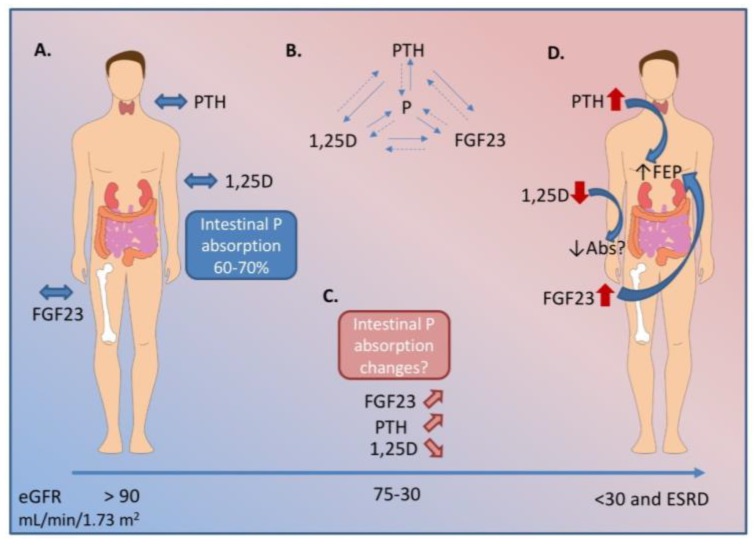
Biochemical changes in hormonal regulators of P (phosphorus) throughout the course of chronic kidney disease (CKD). **A.** In health, fibroblast growth factor 23 (FGF23) from osteocytes, parathyroid hormone (PTH) from the parathyroid gland, and 1,25-dihyxdroxyvitamin D (1,25D) work together to keep serum P within a tight range. A healthy person absorbs 60–70% of P in a mixed diet. **B.** PTH, 1,25D, and FGF23 also regulate each other in a series of feedback loops. **C.** As renal decline progresses, the kidney loses its ability to excrete excess P. FGF23 begins to rise earlier in the disease course, followed by a decline in 1,25D, and a rise in PTH. **D.** In end stage renal disease, the hormonal regulation is unable to maintain serum P within an normal range. At this stage, patients have very high FGF23, PTH, and low 1,25D. The biochemical profile in early and moderate stage CKD indicate intestinal P absorption should be decreased. However, literature suggests P absorption may be inappropriately maintained and, therefore, contributes to overall P burden in CKD. Dashed lines indicate negative feedback. Abs indicates intestinal P absorption; FEP indicates fractional excretion of P from the kidney; eGFR indicates estimated glomerular filtration rate. ESRD indicates end stage renal disease.

Phosphorus homeostasis is controlled by a multi-tissue axis that includes the kidney, intestine, bone, and parathyroid glands [16] as shown in Figure 1A. Dietary phosphorus is absorbed via the intestine, while the bone is the major reservoir for the body’s phosphorus stores, and the kidney filters, reabsorbs, and excretes phosphorus from the body. The parathyroid glands, bone, and kidney are also endocrine organs that produce the main known phosphorus regulating hormones: parathyroid hormone (PTH), fibroblast growth factor 23 (FGF23), and 1,25-dihyxdroxyvitamin D (1,25D). These hormones have complex functions at the tissue level to regulate phosphorus homeostasis, but also regulate one another in a series of negative feedback loops [17]. Figure 1B is a graphical depiction of these feedback loops.

Adults have ~700 g of total body phosphorus, of which ~85% resides in bone as hydroxyapatite, ~14% is intracellular phosphorus, and the remaining ~1% is in extracellular fluid which includes circulating inorganic phosphate [12]. To the best of current knowledge, the primary signal to initiate phosphorus homeostatic mechanisms is an alteration of serum phosphorus, which represents a tiny fraction of total body phosphorus. Indeed, understanding the complex relationship of these hormones is best understood through the lens of these alterations. In response to increased serum phosphorus, the parathyroid glands produce a rapid response by increasing PTH secretion. While PTH primarily acts to maintain calcium homeostasis [18], PTH is also released in response to elevated serum phosphorus [7,17,19]. PTH is a phosphaturic hormone that decreases renal phosphorus reabsorption [16]. PTH also stimulates the renal conversion of 25-hydroxyvitamin D to 1,25D via the CYP27B1 enzyme. The main role of 1,25D in phosphorus homeostasis is to increase intestinal phosphorus fractional absorption [20,21,22]. Both PTH and 1,25D stimulate production of the third main, and most recently discovered phosphorus-regulating hormone, FGF23. FGF23 is produced by osteocytes in bone [17,19,23]. Like PTH, FGF23 is a phosphaturic hormone that regulates serum phosphorus. The main function of this hormone is to promote phosphaturia [19]. This is evidenced by patients who have genetic disorders that cause extremely high serum intact FGF23 levels. These patients exhibit severe renal phosphorus wasting, hypophosphatemia, and rickets [24]. FGF23 and PTH both promote phosphaturia by decreasing expression of phosphorus transporters in the brush border membrane of the nephron [17,25]. However, the decrease in renal phosphorus reabsorption stimulated by FGF23 is independent of PTH action [26] as FGF23 also inhibits PTH [25]. It is important to note that although both PTH and FGF23 promote phosphaturia, FGF23 is a stronger regulator of phosphorus excretion [25] than PTH. When the rapid release of PTH is not sufficient, FGF23 secretion is stimulated by sustained serum phosphorus elevations to cause a strong phosphaturic response. Negative feedback loops between PTH, 1,25D, and FGF23 exist and are critical to the regulation of these hormones’ control over phosphorus homeostasis. While PTH and 1,25D increase FGF23, FGF23 decreases PTH synthesis and secretion as well as decreases renal production of 1,25D [17,18]. PTH stimulates 1,25D production, and 1,25D in turn downregulates itself and PTH [17,25,26,27]. These hormones alter phosphorus homeostasis through the regulation of phosphorus transporters located in the brush border membrane of the renal and intestinal epithelial cells.

## 3. Mechanisms of Intestinal Phosphorus Absorption

What is known about phosphorus absorption is preceded by the discovery of active phosphorus transport in the renal proximal tubules [28]. The kidney expresses type II sodium-dependent phosphate transporters, NaPiIIa and NaPiIIc, which actively reabsorb phosphorus in the proximal tubules. Intestinal phosphorus absorption occurs similarly to renal phosphorus reabsorption in that, like the kidney, the intestine contains an active, sodium-dependent component of phosphorus absorption. The intestine expresses both type II and type III sodium-dependent phosphate transporters and occurs via sodium-dependent (transcellular) pathways [29]. NaPiIIb, analogous to renal transporter NaPiIIa, is the lone type II transporter expressed in the intestine and is encoded by the gene SLC34A2 [29]. NaPiIIb has the highest affinity for divalent phosphate (HPO_4_^−2^), and transports sodium and phosphorus in a 3:1 ratio across the membrane. This transport is made possible by the efflux of sodium on the basolateral side of the cell, leaving a lower concentration of sodium intracellularly [30]. Transcellular transport ultimately produces a net positive charge in the intracellular space. Type III, sodium-dependent, phosphorus transporters PiT-1 and PiT-2 are expressed in the human intestine [29]. PiT transporters cotransport monovalent phosphate (H_2_PO_4_^−1^) in a 2:1 ratio of sodium:phosphate. Although studies have suggested that type III transporters may compensate for intestinal phosphorus absorption in times where type II transporters are compromised [29], in vitro studies of phosphorus absorption performed on murine ileum segments have shown that that NaPiIIb is responsible for a majority (reported >90%) of transcellular phosphorus transport [31,32], but that transcellular transport accounts for only 50% of total phosphorus transport in the intestine [31]. 

The intestine also transports phosphorus in a sodium independent, paracellular manner (also referred to as passive absorption). This is in contrast to the kidney where phosphorus transport is completely negated when sodium is not present in the tubules [28]. This indicates that no paracellular phosphorus transport occurs in the renal tubules. Paracellular phosphate transport is unsaturable and is directly related to the phosphate load in the intestinal lumen [21,33] and is traditionally considered unregulated. However, regulation may yet exist and may particularly involve tight junction proteins such as claudins [29]. On the other hand, there are several known regulators of transcellular sodium-dependent phosphate transport in the intestine, namely 1,25D and dietary phosphorus intake. 

## 4. 1,25-Dihydroxvitamin D Regulation of Intestinal Phosphorus Absorption

The role of 1,25D in the regulation of intestinal phosphorus fractional absorption is well established. Early studies have shown healthy rats and rats repleted with vitamin D show increased phosphate flux and phosphate uptake across the intestinal brush border membrane [20,34]. Specifically, Walling [20] showed that 1,25D repletion of vitamin D deficient rats increased active transport of phosphorus across all segments of the intestine. In accordance with increased phosphorus flux, 1,25D treatment increases gene expression of the type III transporter PiT-2 [34]. In contrast to type III transporters, evidence supports that NaPiIIb expression is post-transcriptionally regulated by 1,25D. Hattenhaur et al. also found that treatment with cholecalciferol did not change NaPiIIb transcription, but did increase NaPiIIb Vmax. These data are consistent with Walling and Katai [20,34] and suggest that 1,25D influences the amount of NaPiIIb protein located in the intestinal brush border membrane where it is active. More evidence to support this non-genomic role of 1,25D in the regulation NaPiIIb is that NaPiIIb does not have a vitamin D response element (VDRE) in its genome [35]. Other studies have suggested that perhaps 1,25D has non-genomic control over intestinal phosphorus absorption, but these studies have not linked the non-genomic action with intracellular signaling pathways that control the NaPiIIb protein [36]. Interestingly, the effect of dietary phosphorus restriction on intestinal phosphorus fractional absorption was previously thought to be a result of decreased systemic 1,25D. However, recent studies have shown that dietary phosphorus restriction has a vitamin D-independent effect on intestinal phosphorus fractional absorption [37]. Details on the physiologic impact of dietary phosphorus load are discussed in the following section.

## 5. Role of Dietary Phosphorus Load in Intestinal Phosphorus Absorption

Dietary phosphorus restriction is another factor known to affect intestinal phosphorus fractional as well as absolute absorption. Because passive phosphorus absorption is dependent on the intestinal luminal phosphorus load, absolute phosphorus absorption is greater with higher levels of dietary phosphorus and lower with lower levels of dietary phosphorus, as described above. The opposite relationship is observed between dietary phosphorus intake level and intestinal phosphorus fractional absorption (i.e., absorption efficiency). An early observation of this was reported by Lee et al. [38]. Young rats were fed a phosphorus restricted diet of 0.03% phosphorus (~1/10 of their dietary phosphorus requirement) for six weeks which resulted in increased jejunal phosphorus fractional absorption. However, this study represents severe dietary phosphorus restriction and therefore these data may not be translatable to “normal” physiology. However, others have since shown that even modest dietary phosphorus restrictions elicit similar responses in phosphorus fractional absorption. Giral et al. [39] showed increased phosphorus uptake into brush border membrane vesicles isolated from the jejunum in young male rats fed a low phosphorus diet (0.1%, ~1/3 of the dietary requirement) compared to higher phosphorus diets (0.6% & 1.0%). Saddoris et al. [40] also showed increased intestinal phosphorus uptake in weanling pigs fed a diet moderately (43%) reduced in phosphorus.

Because of the established effect of 1,25D on increasing phosphorus fractional absorption, and because low phosphorus diets cause an elevation in 1,25D [41,42], it was thought that low phosphorus diets affected phosphorus fractional absorption via a 1,25D mechanism. However, studies in vitamin D receptor (VDR) knockout and CYP27B1 knockout mice have demonstrated that the effect of low phosphorus diets on intestinal phosphate transport is vitamin D-independent. For example, Capuano et al. [37] examined the effect of a 5-day low phosphorus (0.1%) diet on intestinal NaPiIIb messenger RNA (mRNA) and protein levels in male VDR -/-, CY27B1 -/-, and wild type 10–12 weeks old mice. Results showed that the dietary induced changes were similar in wild type mice, VDR -/-, and CYP27B1 -/- mice. The low phosphorus diet increased NaPiIIb gene expression between 3 and 5 fold in all mice regardless of genotype [37]. Similarly, Segawa et al. [43] studied 8-week-old VDR (-/-) mice and found that a low phosphorus diet (0.25%) for 4 weeks induced a greater than two-fold upregulation of NaPiIIb mRNA that corresponded with a one-fold increase in NaPiIIb protein expression. Collectively, these data indicate that intestinal regulation of NaPiIIb and intestinal phosphorus fractional absorption by phosphorus restriction is not dependent on the genomic actions of 1,25D through VDR.

## 6. The Pathophysiological Changes in Phosphorus Homeostasis in Chronic Kidney Disease

### 6.1. CKD is a Progressive Disease Affecting Phosphorus Homeostasis

Because the kidney is the main regulator of phosphorus homeostasis, the decline in kidney function that occurs in CKD affects phosphorus homeostasis. CKD is a progressive disease that is categorized into stages based on estimated glomerular filtration rate (eGFR) ranging from stage 1 (mild) to stage 5 (severe). Alterations in phosphorus homeostasis begin to occur in the early stages of the disease that function to maintain serum phosphate in a normal range until late CKD [2]. Data from a Chronic Renal Insufficiency Cohort study show that the first alteration observed is elevated serum FGF23, followed be decreased serum 1,25D, elevated serum PTH, and eventually elevated serum phosphate. It is important to note that serum phosphate levels are maintained within normal range by the actions of FGF23, PTH, and 1,25D until late CKD, when renal function is so severely impaired that these hormonal compensations are inadequate [25,44,45]. Some have proposed that the early rise in FGF23 may be caused by a deficiency of the FGFR membrane co-receptor, klotho [2,25,26]. Klotho concentration declines with age and with CKD progression [11,14,46]. Klotho deficiency leads to FGF23 resistance and continual secretion of FGF23 from osteocytes [25]. It is proposed that FGF23 might then develop affinities for other FGFRs that do not require klotho as a co-receptor (i.e., FGFR4 in the heart) and stimulate other off-target tissues [25,47]. Elevated serum phosphate is associated with vascular calcifications and death in patients with CKD [48]. So, these hormonal alterations are necessary to control serum phosphate. However, these alterations are not without consequence. For example, elevated FGF23 has been shown to directly induce left ventricular hypertrophy in mice, which supports observations of greater left ventricular mass with higher levels of FGF23 in patients with CKD [47]. 

### 6.2. Phosphorus Management in CKD is Challenged by Insufficient Clinical Management Strategies

A major strategy in the clinical management of CKD-MBD is to decrease phosphate load by impairing intestinal phosphorus absorption. This includes use of dietary phosphorus restriction as well as phosphate binder medications. A combination of these two therapies is often required for phosphorus control in CKD, but also becomes insufficient as renal function declines [12]. Initiation of phosphorus restriction typically occurs in moderate stage CKD (stage 3 and 4) when serum phosphorus is slightly elevated, but still within the normal range or during stage 5 when serum phosphorus is greater than 5.5 mg/dL [6]. Dietary phosphorus may also be restricted when serum PTH levels become elevated past target ranges for a specific CKD stage [6].

Dietary phosphorus restriction is not easily achieved or maintained. Compliance is difficult for patients because of the difficult balance between achieving adequate protein and low phosphorus. Phosphorus is contained in many naturally occurring forms in the American diet as well as in many food additives [13,49]. Thus, actual intake of phosphorus in CKD patients is often higher than the KDOQI recommendations [50,51]. Not only is the ubiquity of phosphorus in the diet problematic, but the high bioaccessibility (estimated at 90–100%) of phosphorus-containing food additives contributes to a higher phosphorus burden compared with naturally occurring forms in plant and animal foods [52,53]. It is conceivable that there would be variation in % bioaccessibility even among the inorganic phosphate salts depending on cation (e.g., Na^+^, K^+^, Ca^2+^), especially for those salts that are less soluble (e.g., calcium phosphates); however, to our knowledge, this has not been evaluated. Thus, to date, the inorganic phosphates found in food additives have largely been considered as a single entity. Because of their estimated high bioaccessibility, limiting phosphorus-containing food additives has emerged as a novel treatment strategy to reduce phosphorus burden in patients with CKD. Sullivan et al. [54] demonstrated that an educational intervention by a registered dietitian to teach hemodialysis patients how to avoid consuming phosphorus-containing food additives had a significant effect on reducing serum phosphate over the 3-month study compared with that of standard of care. This was corroborated recently by de Fornasari et al. [55] who also showed a significant reduction in serum phosphate over 3 months in adult hemodialysis patients who were randomized to a similar education intervention to reduce phosphorus-containing food additives. Importantly, this reduction in serum phosphate was achieved without affecting markers of nutritional status. There is also new randomized controlled trial evidence [56] that high dietary phosphorus intake from highly-bioaccessible phosphorus-containing food additives increases blood pressure in healthy adults, which may imply a role of phosphorus metabolism in the prevalent etiology of hypertension leading to CKD as well as CKD-exacerbated hypertension. The concept of utilizing the knowledge of phosphorus bioaccessibility to improve phosphorus status in patients with CKD has also been applied to interventional studies of plant-based versus meat-based diets [47,57]. These studies have shown that switching from meat-based diets with higher phosphorus bioaccessibility to plant-protein based diets with lower bioaccessibility can lower serum phosphate or reduce 24-h urine phosphorus excretion (reflecting reduced absolute phosphorus absorption in response to a controlled intervention). These approaches, limiting phosphorus-containing food additives and/or shifting to a plant-protein based diet, are particularly of interest as they offer an opportunity to potentially reduce phosphorus burden without sacrificing protein intake [58]. 

Phosphate binders offer another commonly-used strategy to prevent the absorption of dietary phosphorus. Phosphorus binders are considered to be the second-line therapy when dietary restriction of phosphorus is insufficient at lowering serum phosphorus [6]. There are many different types of binders with varying binding capacities of phosphorus [6]. These are broadly categorized as calcium-based and non-calcium-based. There is ongoing debate [59,60] regarding the use of calcium-based phosphate binders in light of observations that they may cause calcium retention [61,62] and vascular calcifications [63].

Compliance with phosphate binders is also a problem due to pill burden and various side effects [50]. Several types of phosphate binders are available and are generally categorized as calcium-based and non-calcium-based. All phosphate binders on the market have been shown to be effective at reducing serum phosphate at least in short-term studies [64]. Several factors like the size and number of pills, gastrointestinal side-effects, and ability to chew (needed for lanthanum carbonate) must be taken into consideration for an individual patient. A recent review on phosphate binders gives a comprehensive overview and comparison of available binders [65]. Overall, the binding capacity of any phosphate binder has its limits where these alone cannot fully negate phosphorus surplus by late stage CKD. Dialysis patients experience a 300–500 mg/day phosphorus surplus if consuming 900–1500 mg/day of phosphorus [50] while phosphorus binders have a binding capacity of approximately 250 mg/day. This excess phosphorus, if absorbed, can contribute to hyperphosphatemia [50]. Thus, many CKD patients are unable to achieve targets for serum phosphorus [50]. Because of inability of the kidney to sufficiently excrete phosphorus, the intestine is a logical target for therapies aimed at reducing phosphorus burden. However, a better understanding of intestinal phosphorus absorption mechanisms, factors affecting absorption, and how CKD is a modifying factor are needed to develop better strategies for phosphorus management.

## 7. Intestinal Phosphorus Absorption in the Context of CKD 

Classic phosphorus homeostasis would predict a decrease in intestinal phosphorus fractional absorption in CKD, namely in response to elevated FGF23 and subsequent decrease in 1,25D; i.e., an intestinal compensation for renal decline. In patients with very severe CKD, it appears that intestinal phosphorus fractional absorption is indeed reduced compared with healthy controls [66]. However, studies using CKD animal models suggest an absent or insignificant intestinal adaptation to reduce phosphorus burden in CKD. Marks et al. [41] showed that 5/6 nephrectomized (CKD) rats had significantly lower 1,25D, higher PTH, and higher serum phosphorus levels that sham-operated rats [41], a biochemical scenario consistent with late stage CKD in humans. However, fractional phosphorus absorption assessed by in situ ligated loops in the jejunum and duodenum was not statistically different between the CKD rats and control rats, and was inconsistent with the hormonal changes observed [41]. Additionally, there was no change of NaPiIIb mRNA in CKD rats compared to controls. 

Loghman-Adham [67] et al. modeled a moderate stage CKD by inducing renal failure via a two-part 5/6 nephrectomy procedure and studied the animals when they exhibited increased plasma creatinine and blood urea nitrogen (BUN) without significant increases in plasma phosphorus. Control and CKD rats were fed either a low phosphorus diet (0.07%) or a high phosphorus diet (0.95% for 5 days). Results showed that the low phosphorus diet increased phosphorus uptake in brush border membrane vesicles by over two-fold in the CKD and control rats alike. Consistent with the change in phosphorus uptake, kinetic analysis showed the V_max_ for NaPiIIb was increased by almost three-fold in both control and CKD rats when fed a low phosphorus diet. These animal data suggest that the intestine’s ability to adapt to dietary changes in phosphorus may be preserved in the context of moderate renal failure, and intestinal phosphorus fractional absorption may not be decreased with decline in kidney function, suggesting a break in the homeostatic regulation of phosphorus in CKD. However, much of the animal literature is limited to studies using in vitro or ex vivo methods of phosphorus absorption assessment. Further, few human studies of direct measurement of intestinal phosphorus absorption exist, particularly in CKD. However, studies that have used 24-h urine phosphorus excretion as a proxy for phosphorus absorption in the context of controlled feeding have shown that patients with moderate CKD have similar 24-h urine phosphorus excretion compared with healthy controls [62]. However, 24-h urine phosphorus may be flawed as a biomarker for intestinal phosphorus absorption as demonstrated from phosphorus balance studies in moderate-stage CKD patients [68], and if lower 24-h urine phosphorus is observed in CKD patients, it may actually reflect greater phosphorus retention, rather than reduced absorption. Thus, studies of utilizing direct measures of phosphorus absorption are needed to determine how phosphorus absorption is affected by CKD and to develop translatable strategies to decrease phosphorus absorption in early chronic kidney disease. 

## 8. Summary and Conclusions

In normal physiology, phosphorus homeostasis involves a complex, multi-tissue axis where the kidney is the main regulator. The kidney and the intestine share similarities in their regulation of phosphorus handling. 1,25D regulates the main known intestinal phosphorus transporter, NaPiIIb, and dietary phosphorus restriction is an important factor affecting intestinal phosphorus absorption, independent of 1,25D. When kidney function begins to decline, there is hormonal compensation of increased FGF23 and PTH, and decreased 1,25D. The main effect of these alterations is to increase fractional phosphate excretion at the kidney. However, adverse cardiovascular and bone effects are collateral damage from this compensation. With the kidney progressively failing to regulate phosphate homeostasis, limiting intestinal absorption becomes a primary focus. However, intestinal absorption of phosphorus may be maintained at normal levels in CKD, at least until very late in the disease, despite decreased 1,25D, which should reduce fractional absorption. This would imply a lack of response or compensation by the intestine. A better understanding of mechanisms of intestinal phosphorus absorption in the context of CKD is necessary to improve on existing therapies for phosphate management.
